# Plasma-derived mitochondria as a minimal manipulation alternative for attenuating inflammatory immune responses

**DOI:** 10.1093/rb/rbaf132

**Published:** 2025-12-26

**Authors:** Seong-Hoon Kim, Eun-Seo Back, Ikhyun Lim, Mina Lim, Mi Jin Kim, Kyunghoon Min, Chang-Koo Yun, Yong-Soo Choi

**Affiliations:** Department of Bio-Convergence Science, Graduate School, CHA University, Seongnam 13488, Republic of Korea; Department of Life Sciences, Graduate School, CHA University, Seongnam 13488, Republic of Korea; Department of Bio-Convergence Science, Graduate School, CHA University, Seongnam 13488, Republic of Korea; Department of Bio-Convergence Science, Graduate School, CHA University, Seongnam 13488, Republic of Korea; Department of Life Sciences, CHA University, Seongnam 13488, Republic of Korea; Department of Rehabilitation Medicine, CHA Bundang Medical Center, CHA University School of Medicine, Seongnam 13496, Republic of Korea; Department of Life Sciences, CHA University, Seongnam 13488, Republic of Korea; Department of Bio-Convergence Science, Graduate School, CHA University, Seongnam 13488, Republic of Korea; Department of Life Sciences, Graduate School, CHA University, Seongnam 13488, Republic of Korea; Department of Life Sciences, CHA University, Seongnam 13488, Republic of Korea

**Keywords:** inflammation modulation, macrophage polarization, minimal manipulation, plasma-derived mitochondria, platelet-derived mitochondria

## Abstract

Platelet-rich plasma (PRP) has long been used to promote tissue repair through its content of cytokines, growth factors and platelet-derived mitochondria (PLT-mito). While effective, PRP often triggers an early M1-type inflammatory response that may worsen symptoms in chronic inflammatory conditions and limit its clinical utility. Harvesting functional PLT-mito usually requires platelet activation or mechanical disruption, which can exceed minimal manipulation thresholds under regulatory guidelines. In contrast, plasma-derived mitochondria (p-mito) provide an alternative, as they are naturally present in circulating plasma and can be obtained by simple centrifugation without activation or cell disruption. In this study, we compared the effects of platelets, PLT-mito, platelet releasate and p-mito on macrophage polarization and fibroblast repair assays using THP-1 and HDF models. Macrophage polarization was quantified at the RNA level by PCR of M1-associated (CD80, CD86) and M2-associated (CD163, CD206) surface-marker transcripts after each treatment. Platelets and platelet releasate predominantly induced M1-like polarization, whereas both PLT-mito and p-mito promoted an M2 phenotype. Notably, p-mito achieved M2 induction comparable to PLT-mito without requiring prior activation or manipulation. In a fibroblast scratch migration assay, p-mito enhanced cell migration and proliferation, replicating the pro-reparative effects of platelets. These findings suggest that p-mito are a functionally competent, cell-free therapeutic modality capable of modulating macrophage phenotypes without triggering early inflammatory priming. This minimally manipulated, clinically accessible mitochondrial therapeutic can be prepared and applied in a manner similar to PRP, with the potential to reduce early pro-inflammatory responses.

## Graphical abstract

**Figure:**
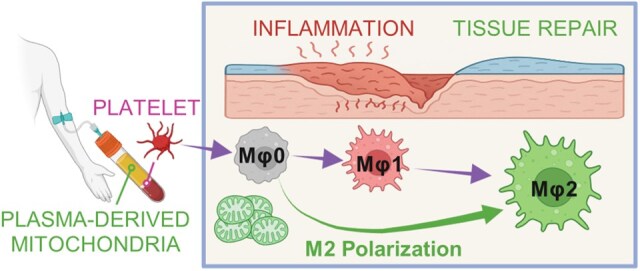

## Introduction 

Mitochondria are essential organelles that generate adenosine triphosphate (ATP), regulate reactive oxygen species and orchestrate cellular metabolism, survival and intercellular signaling [1]. Recent studies have revealed that mitochondria are not static within their cells of origin, but can be actively transferred between cells to modulate immune responses and metabolic recovery [2–5]. This intercellular mitochondrial transfer has been described in diverse physiological and pathological contexts, including immune regulation, inflammation control, host defense and response to injury [2–6]. Among the various donor cell types, platelets have recently attracted attention as a readily available and clinically accessible source of functional mitochondria [5, 7, 8].

Platelets, traditionally recognized for their role in hemostasis and as reservoirs of growth factors and cytokines, have now emerged as natural carriers of functional mitochondria [7–10]. This mitochondrial transfer, achieved through direct cell contact or extracellular vesicles [11, 12], enables platelets to deliver healthy mitochondria to recipient cells at sites of injury or inflammation [9, 13]. Beyond restoring bioenergetic capacity, such transfer may reprogram recipient cells toward anti-inflammatory phenotypes, providing a novel mechanism that expands the immunomodulatory functions of platelets beyond their canonical roles [7–9, 11, 13].

Despite these advances, the clinical application of platelet-rich plasma (PRP) and platelet-derived mitochondrial (PLT-mito) therapies is limited by several key challenges [14–17]. The beneficial effects of PRP have largely been attributed to its rich content of bioactive proteins, yet in chronic inflammatory settings, PRP can exacerbate symptoms such as pain, swelling and discomfort, restricting its use [14–16]. Furthermore, the extraction of functional PLT-mito from platelets typically necessitates *ex vivo* activation or physical disruption, which exceeds current minimal manipulation guidelines and complicates standardization for broad clinical implementation [18]. These limitations highlight the need for novel, minimally manipulated and clinically scalable mitochondrial therapeutics.

While PLT-mito have been increasingly investigated for their roles in modulating inflammation and, secondarily, promoting tissue repair [19, 20], recent advances have identified bioactive, cell-free mitochondria naturally present in human plasma, termed plasma-derived mitochondria (p-mito) [21, 22]. Unlike PLT-mito, which require platelet activation or mechanical disruption for isolation, p-mito can be obtained by simple centrifugation without cellular activation or lysis [21, 22], thus, qualifying as a minimally manipulated product [23]. Our recent work demonstrated that p-mito can be reproducibly isolated from donors across a wide age range and with diverse clinical backgrounds, yielding consistent quantity and preserved bioenergetic function [24]. However, whether p-mito exerts immunomodulatory effects comparable to PLT-mito, and whether their underlying mechanisms differ, remains unknown. Addressing this gap is essential for defining the translational potential of p-mito. Clarifying these differences could guide the development of safer and more targeted mitochondrial therapeutics.

In conventional platelet-based approaches, macrophages typically undergo an initial pro-inflammatory M1 phase, marked by high expression of iNOS and secretion of TNF-α, IL-1β and other inflammatory mediators [25–27], followed by a delayed transition to the anti-inflammatory, pro-repair M2 phenotype [25, 27]. We hypothesized that p-mito could attenuate or shorten this initial M1-skewed phase, favoring a more direct shift toward M2-like macrophage polarization without pronounced early inflammatory priming. Validating this mechanism would position p-mito as a practical, inflammation-free alternative to platelet-based therapies, particularly for patients with chronic inflammatory conditions. To test this hypothesis, we compared the effects of platelets, PLT-mito, platelet releasate and p-mito on macrophage polarization and fibroblast-mediated wound closure. This comparison will determine whether p-mito can overcome the inflammatory limitations of conventional platelet therapies while offering a scalable, minimally manipulated solution for immune modulation.

## Materials and methods

### Blood collection and ethics approval

Peripheral blood samples were obtained into ethylenediaminetetraacetic acid-coated tubes from healthy adult volunteers (*n* = 10; ages 24–55; 2 female, 8 male) at CHA University Hospital (Seongnam, Korea). All donors with a history of chronic autoimmune, metabolic or infectious disease were excluded. Written informed consent was acquired before blood collection, and study procedures were approved by the Institutional Review Board (IRB No. 2021-06-044). Samples were processed within 1 h after collection to ensure cell viability.

### Platelet isolation

Platelets were isolated by differential centrifugation. Whole blood was centrifuged at 1000 ×*g* for 5 min at room temperature to obtain PRP. PRP was further centrifuged at 2000 × *g* for 10 min to pellet platelets, which were washed twice in Dulbecco’s phosphate buffered saline (DPBS) and subsequently resuspended in SHE buffer (250 mM sucrose, 20 mM HEPES, 2 mM EGTA, 10 mM KCl, 1.5 mM MgCl_2_, pH 7.4) for downstream experiments.

To generate damaged platelets, platelets were pre-treated with 100 μM carbonyl cyanide m-chlorophenyl hydrazone (CCCP) for 1 h at room temperature. CCCP-treated platelets were washed twice in DPBS.

### Mitochondrial isolation from platelets and plasma

For isolated PLT-mito, platelets were incubated with 1 mg/mL CaCl_2_ for 2 h at room temperature to induce activation and mitochondrial release. After activation, suspensions were centrifuged at 2000 × *g* for 10 min at 4°C to remove debris, and then, the supernatant was centrifuged at 20 000 × *g* for 10 min to pellet the mitochondria. The pellet was resuspended in SHE buffer. Platelet releasate (remaining supernatant) was collected and stored at 4°C.

For mechanical isolation of PLT-mito, platelets were disrupted by repeated passage through a 26-gauge syringe needle. Lysates were cleared of debris by centrifugation at 2000 × *g* for 10 min, and mitochondria were isolated as described above.

p-mito were isolated from human plasma obtained after platelet removal, as described in section platelet isolation. Plasma samples were directly centrifuged at 20 000 × *g* for 10 min without any activation or lysis steps, ensuring minimal manipulation. The resulting mitochondrial pellet was washed once with DPBS and resuspended in SHE buffer for immediate downstream analyses.

### Mitochondrial functional assay

The functional characteristics of isolated mitochondria were assessed by measuring protein concentration, respiratory enzyme activity, ATP synthesis and citrate synthase activity.

Mitochondrial protein concentration was measured using the Pierce BCA Protein Assay Kit (Thermo Fisher Scientific, Waltham, MA, USA).

Complex I + III activity was determined by incubating 2 μg of mitochondrial protein in 200 μL reaction buffer (2.5 mM potassium phosphate, pH 7.5; 0.1% BSA; 0.3 mM KCN; 0.05 mM oxidized cytochrome c). After 2 min, 0.2 mM NADH was added to start the reaction and the increase in absorbance at 550 nm was monitored for 20 min using a Synergy HTX microplate reader (BioTek Instruments, Winooski, VT, USA).

Complex IV activity was assessed by mixing 2 μg mitochondria with 200 μL of 25 mM potassium phosphate (pH 7.0) containing 0.05 mM reduced cytochrome c, and measuring the decrease in absorbance at 550 nm for 20 min.

ATP synthesis was evaluated by incubating mitochondria (10 μg protein) with 5 mM ADP for 45 min. ATP generation was measured by mixing an equal volume of CellTiter-Glo 2.0 reagent (Promega, Madison, WI, USA) and detecting luminescence.

Citrate synthase activity was determined by suspending 2 μg mitochondria in 100 mM Tris-HCl buffer (pH 8.0) containing 0.1% Triton X-100, 0.3 mM acetyl-CoA, 0.5 mM oxaloacetate and 0.1 mM DTNB and monitoring absorbance at 412 nm for 20 min. Heat-inactivated p-mito (100°C, 20 min) served as a negative control.

### Mitochondrial transfer and fluorescence imaging

Platelets or isolated mitochondria were labeled with MitoTracker Red CMXRos (Invitrogen) following manufacturer’s instructions and added to THP-1 macrophages labeled with MitoTracker Green FM (Invitrogen). Uptake and localization were visualized on a Ts2 Eclipse inverted fluorescence microscope (Nikon, Tokyo, Japan) using Optiview software (Korea Lab Tech, Hwaseong, Korea).

### Macrophage differentiation and polarization assays

THP-1 monocyte cells (TIB-202; ATCC, Manassas, VA, USA) were maintained in RPMI-1640 medium with 10% fetal bovine serum (FBS) and 0.05 mM 2-mercaptoethanol at 37°C, 5% CO_2_. Cells were differentiated into macrophages with 25 nM phorbol 12-myristate 13-acetate (PMA) for 48 h. Differentiated macrophages were treated for 48 h with platelets (5 × 10^6^ platelets/well), PLT-mito (5 μg protein/well), platelet releasate (50 μL/well or volume equivalent) or p-mito (5 μg protein/well). Cytokine controls (LPS, IFN-γ, IL-4, IL-13) were each added at 20 ng/mL.

### Conventional PCR and gel quantification

Total RNA was isolated using the Easy-spin Total RNA Extraction Kit (iNtRON Biotechnology, Seongnam, Korea) according to the manufacturer’s instructions. cDNA was synthesized from 1 μg RNA with the Maxim RT Premix Kit (iNtRON Biotechnology). PCR was performed using i-StarMAX II PCR Master Mix (iNtRON Biotechnology) and gene-specific primers (listed in Table 1). PCR products were separated by 1% agarose gel electrophoresis, visualized under UV illumination and digitally imaged. Band intensities were quantified by ImageJ software (National Institutes of Health, Bethesda, MD, USA), normalized to β-actin and expressed as percentages relative to the maximum intensity band.

**Table 1:** Primer sequences used for RT-PCR.

Target gene	Forward primer	Reverse primer
CD11b	TCC CGG AAA ACT CAG AGG TC	ACG GGA TGT CAC ACT GGA TT
CD80	GGA AGT GCC CTG GTC TTA CT	TAA CGT CAC TTC AGC CAG GT
CD86	ACG CGG CTT TTA TCT TCA CC	CTT GGC CCA TAA GTG TGC TC
CD163	ATC ATG CTG AGG ATG CTG GA	CCT GCA AAC CAC ATC AGC TT
CD206	TTA GGT GGA GAG GCA GTT GG	CCC GAT CCC TTG TAG AGC AT
β-actin	GCA TCC TCA CCC TGA AGT A	CTG GGG TGT TGA AGG TCT

### Western blotting

For mitochondrial purity assessment, equal amounts of protein from each fraction were denatured with SDS-PAGE loading buffer (LPS Solution, Daejeon, Korea). Proteins were separated by SDS-PAGE and transferred to PVDF membranes. Membranes were blocked in 5% bovine serum albumin and incubated overnight with primary antibody COX IV (Cell Signaling Technology, Danvers, MA, USA; 4844 s), TOM20 (Santa Cruz Biotechnology, Dallas, TX, USA; sc-17764), LAMP-1 (Santa Cruz; sc-20011) and β-actin (Santa Cruz; sc-47778). After washing, membranes were incubated with HRP-conjugated secondary antibodies (Santa Cruz; sc-516102, sc-2357). Signals were developed using the Amersham ECL kit (Cytiva, Marlborough, MA, USA) and captured on a LAS-4000 imaging system (Fujifilm, Tokyo, Japan).

### Fibroblast migration and cell cycle assays

Human dermal fibroblasts (HDFs; PCS-201-012; ATCC) were cultured in DMEM with 10% FBS at 37°C, 5% CO_2_. For scratch migration assays, confluent HDF monolayers were scratched with a sterile pipette tip and treated with platelets (1.5 × 10^7^ platelets/well), p-mito (15 μg protein/well) or controls; phase-contrast images were acquired at 0 h and 24 h using a CKX41 inverted microscope (Olympus, Tokyo, Japan). Wound areas were quantified in ImageJ using the MRI Wound Healing Tool macro, and percent closure was calculated as: %closure = (area_0 h—_area_24 h_)/area_0 h_ × 100. For cell cycle assays, cells were harvested, washed with DPBS and fixed by dropwise addition of 70% ethanol for 30 min. Fixed cells were resuspended in DPBS containing 50 μg/mL propidium iodide and 200 μg/mL RNase A, then incubated at 37°C for 45 min in the dark. Samples were analyzed on a CytoFLEX flow cytometer (Beckman Coulter, Brea, CA, USA), and cell-cycle phase distributions were determined using FlowJo v10.7.1 (BD Biosciences, Franklin Lakes, NJ, USA).

### Statistical analysis

All experiments were performed in triplicate unless otherwise noted. Data are presented as mean ± standard deviation (SD). Statistical analysis was performed using GraphPad Prism 10 (GraphPad Software, San Diego, CA, USA). Group comparisons were carried out using one-way ANOVA with Tukey’s *post hoc* test. A *P* value of <0.05 was considered statistically significant.

## Results

### Platelet-induced M1 polarization of THP-1-derived macrophages

**Figure 1:**
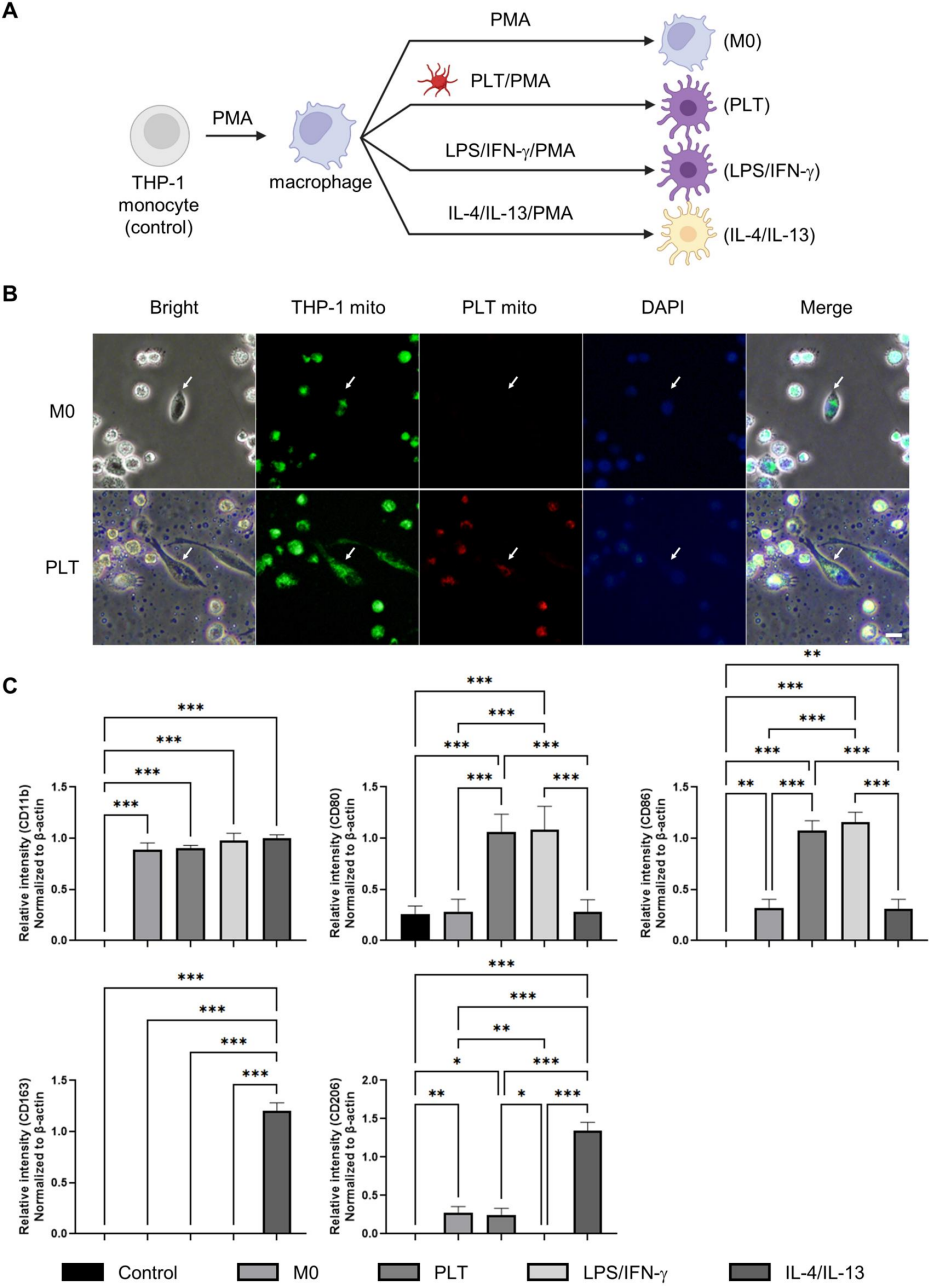
Platelet promotes M1 macrophage polarization. (**A**) Experimental scheme for differentiating THP-1 monocytes into M0 macrophages with PMA, followed by treatment with platelet (PLT), LPS/IFN-γ (M1 control) or IL-4/IL-13 (M2 control). (**B**) Representative fluorescence microscopy images showing recipient THP-1 macrophages containing donor PLT-mito. THP-1 mito and PLT-mito were labeled using MitoTracker dyes, and nuclei were visualized using DAPI staining. Arrows indicate representative cells. Scale bar = 30 µm. (**C**) Analysis of surface markers for M0 (CD11b), M1 (CD80, CD86) and M2 (CD163, CD206) phenotypes. Control denotes PMA-untreated THP-1 monocytes. Data are expressed as normalized RT-PCR band intensities (*n* = 3). *P < 0.05, **P < 0.01, ***P < 0.001.

We first examined whether platelets influence macrophage polarization. THP-1 monocytes were differentiated into M0 macrophages using PMA and then treated with platelets or classical cytokine controls (Figure 1A). Fluorescence microscopy revealed red-labeled donor mitochondria within green-labeled recipient macrophages, confirming mitochondrial transfer from platelets to THP-1 cells (Figure 1B). Platelet exposure selectively increased the expression of M1-associated markers CD80 and CD86, while M2-associated markers CD163 and CD206 remained unchanged, indicating a predominant M1-polarizing effect (Figure 1C).

### Differential effects of platelet-derived immunomodulators and mitochondria on macrophage polarization

**Figure 2:**
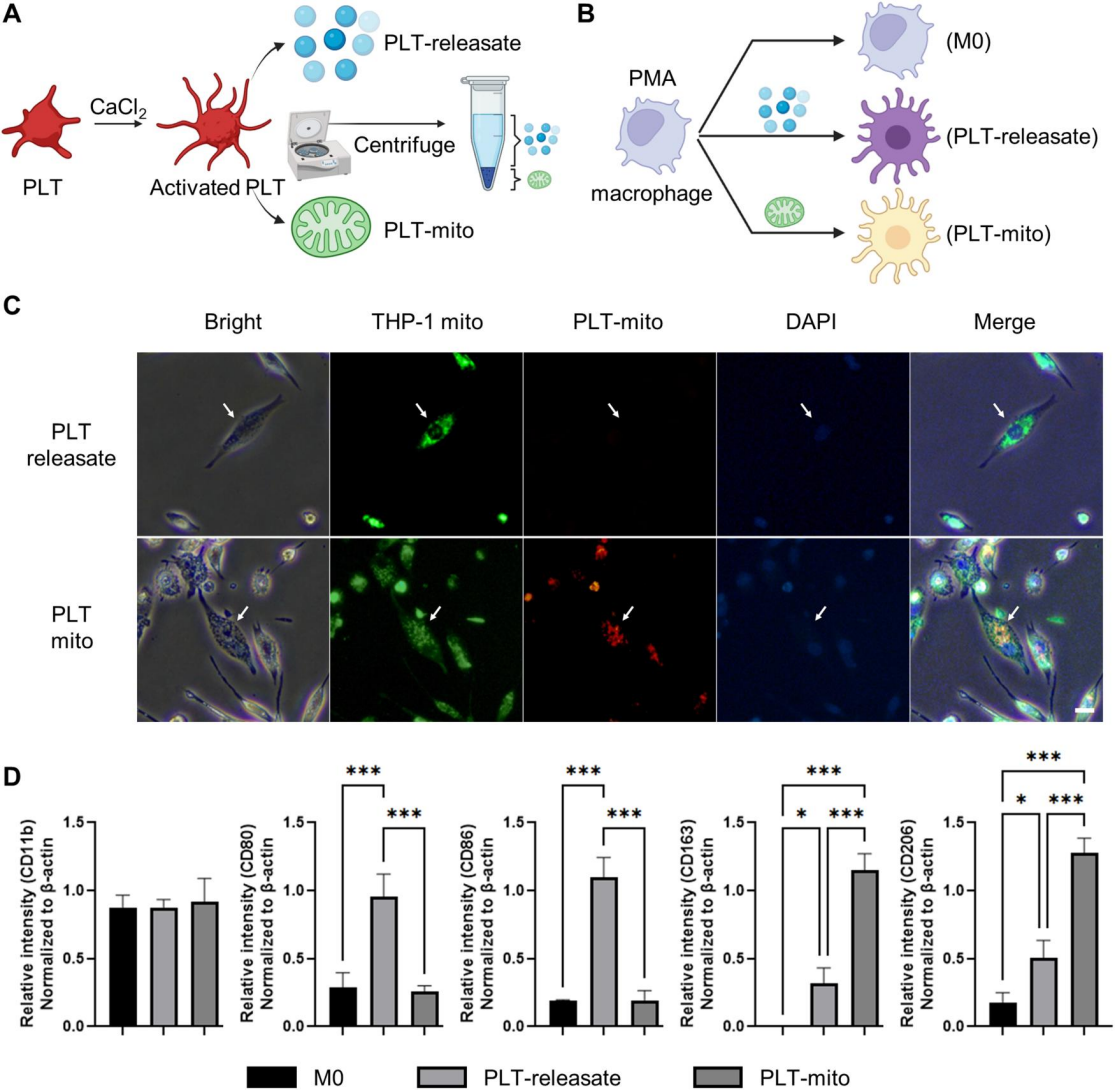
Differential effects of platelet-derived releasate and mitochondria on macrophage polarization. (**A**) Schematic of PLT fractionation into a mitochondrial pellet (PLT-mito) and soluble releasate (PLT-releasate) after activation with CaCl_2_. (**B**) Experimental design for treating PMA-differentiated THP-1 macrophages (M0) with each fraction. (**C)** Representative fluorescence microscopy images showing uptake of exogenous mitochondria (PLT-mito) in PLT-mito–treated THP-1 macrophages, but not in PLT-releasate–treated THP-1 macrophages. Nuclei are visualized using DAPI staining. Arrows indicate representative cells. Scale bar = 30 µm. (**D**) Analysis of M0 (CD11b), M1 (CD80, CD86) and M2 (CD163, CD206) markers after 48 h treatment. Data are expressed as normalized RT-PCR band intensities (*n* = 3). *P < 0.05, **P < 0.01, ***P < 0.001 (*n* = 3).

To dissect the individual contributions of soluble immunomodulators and mitochondria from PLTs in shaping macrophage phenotype, activated platelets were fractionated into PLT-mito and soluble factors (PLT-releasate; Figure 2A). Each fraction was applied separately to PMA-differentiated THP-1 macrophages (Figure 2B).

Fluorescence microscopy confirmed the uptake of red-labeled exogenous mitochondria in PLT-mito–treated cells, whereas no mitochondrial signal was detected in PLT-releasate–treated cells (Figure 2C). Flow cytometric analysis revealed that PLT-releasate markedly increased the M1-associated markers CD80 and CD86, while PLT-mito selectively upregulated the M2-associated markers CD163 and CD206 (Figure 2D). These findings indicate a dichotomous role of platelet-derived components, with soluble immunomodulators promoting M1 polarization and mitochondria favoring M2 polarization.

To determine whether the M2-inducing activity required functional mitochondria, platelets were pretreated with CCCP to dissipate the mitochondrial membrane potential. Because CCCP-treated platelets do not undergo activation, and therefore, fail to release mitochondria or soluble factors [28], mitochondria were instead obtained by mechanical disruption (Supplementary Figure S1A and B). These damaged mitochondria (dPLT-mito) failed to elevate M2 marker expression compared with untreated controls (Supplementary Figure S1C), confirming that intact and functional mitochondria are essential for driving M2 macrophage polarization.

### Long-term phenotypic shifts following platelet treatment

**Figure 3:**
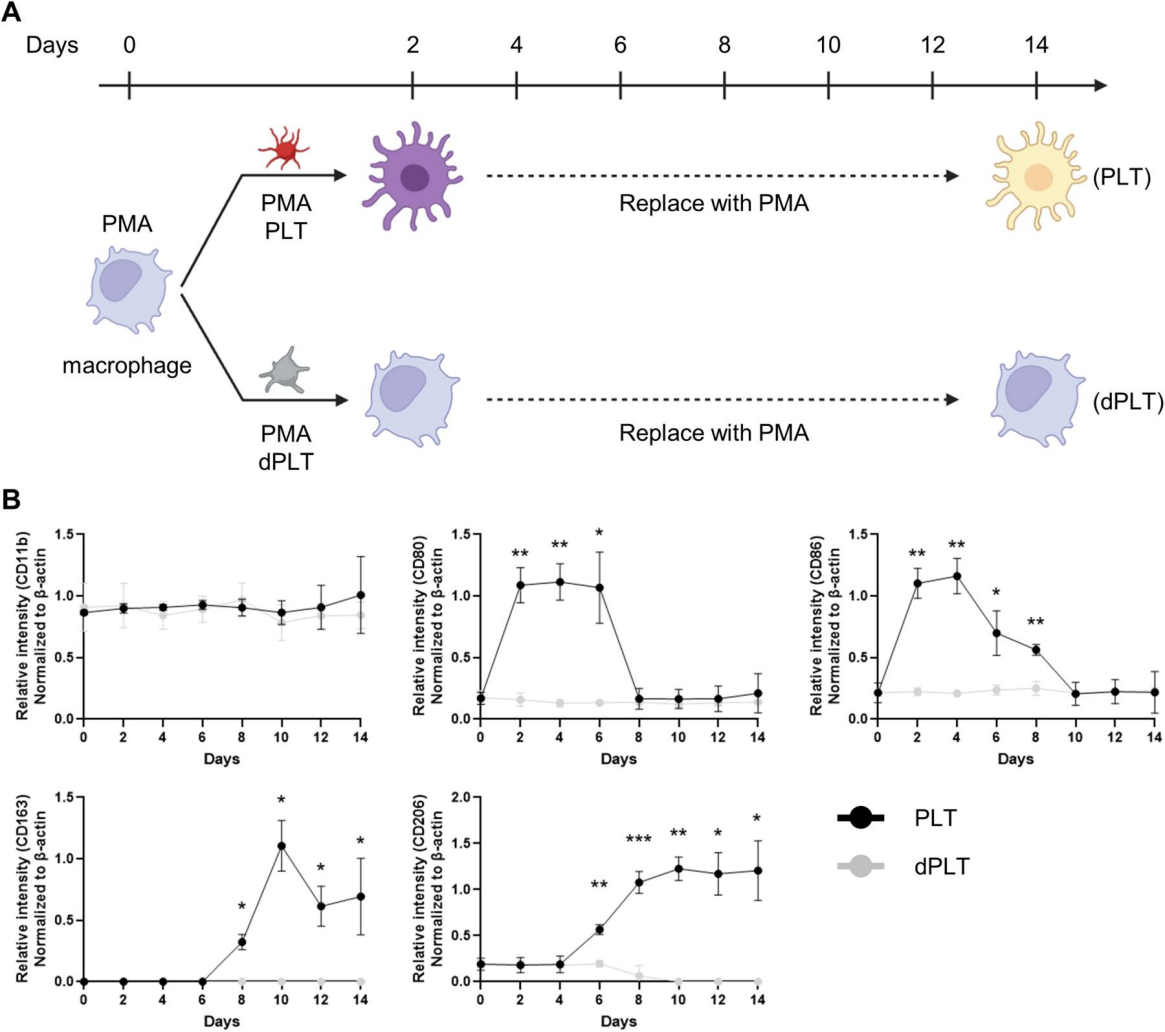
Time-course effects on intact versus damaged platelets on macrophage polarization. (**A**) Experimental timeline. THP-1 cells were differentiated with PMA and then exposed to intact platelets (PLT) or CCCP-treated platelets (dPLT) for the initial period; thereafter, medium was replaced with PMA-containing medium (no platelets) and cells were followed for up to 14 days. (**B**) Marker expression of M0 (CD11b), M1 (CD80, CD86) and M2 (CD163, CD206). Data are expressed as normalized RT-PCR band intensities (*n* = 3). *P < 0.05, **P < 0.01, ***P < 0.001.

To assess the persistence of platelet-induced macrophage phenotypes, THP-1-derived M0 macrophages were treated for 48 h with either intact platelets or CCCP-treated platelets (dPLT; lacking functional mitochondria and releasate), followed by thorough washing to remove residual factors (Figure 3A). Cells were then cultured in PMA-containing medium for up to 14 days with routine media replacement.

In the platelet-treated group, M1-associated markers (CD80, CD86) peaked at Day 4 and gradually declined thereafter, while M2-associated markers (CD163, CD206) began to increase from Day 6 and remained elevated through Day 14 (Figure 3B). Between-group comparisons at matched time points (PLT vs dPLT) were significant at the indicated days. dPLT remained near baseline across the time course, indicating that functional mitochondria are required for the transition from an initial M1 response to a sustained M2 phenotype.

### Comparative analysis of platelet-derived and plasma-derived mitochondria on macrophage polarization

We first validated the purity of PLT-mito and p-mito by Western blotting. Mitochondrial markers COX IV and TOM20 were detected in both PLT-mito and p-mito fractions, whereas lysosomal (LAMP1) and cytoskeletal (β-actin) proteins were observed only in the PLT-mito fraction, confirming the high purity of p-mito preparations (Supplementary Figure S2).

**Figure 4:**
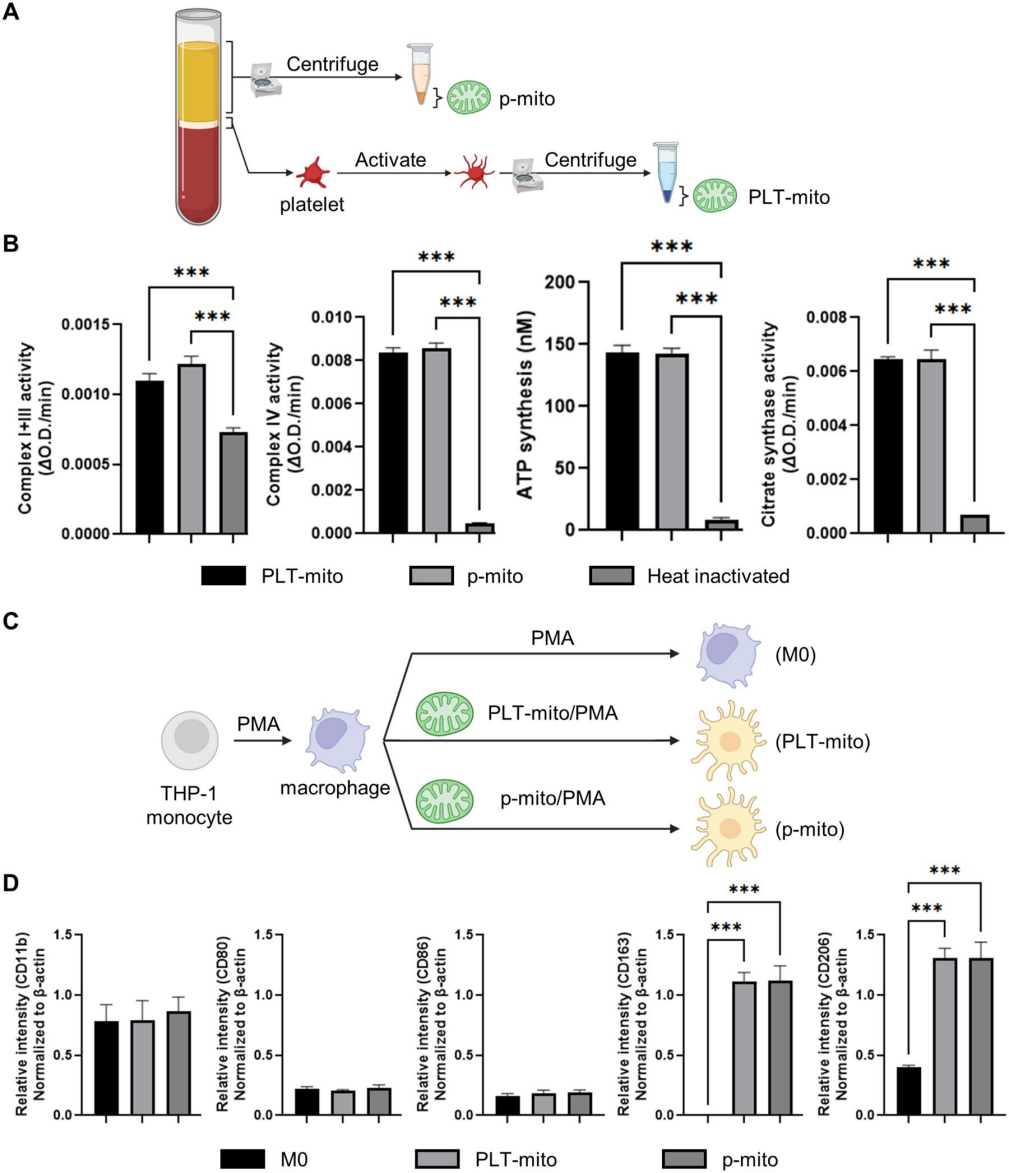
Comparative mitochondrial characteristics and macrophage polarization induced by PLT-mito and p-mito. (**A**) Schematic showing isolation of PLT-mito and p-mito. (**B**) Mitochondrial functional assays for complexes I + III, complex IV, ATP synthesis and citrate synthase activity, normalized to maximum activity. (**C**) Experimental design for treating THP-1-derived macrophages with PLT-mito or p-mito. (**D**) Marker analysis of CD11b (M0), CD80/CD86 (M1) and CD163/CD206 (M2) expression in macrophages. Data are presented as mean ± SD (*n* = 3); ***P < 0.001.

Mitochondrial functional assays revealed comparable activities of complexes I+III and IV, ATP synthesis and citrate synthase between the two mitochondrial sources (Figure 4B). When introduced to THP-1-derived macrophages, both PLT-mito and p-mito induced similar upregulation of M2 markers CD163 and CD206, with no significant differences in M0 (CD11b) or M1 (CD80, CD86) marker expression (Figure 4C and D). These data support comparable bioenergetic properties and directionally similar immunoregulatory effects, consistent with an M2-associated response *in vitro*.

### Plasma-derived mitochondria reproduce platelet-driven fibroblast repair responses

Platelets enhance tissue repair via growth factors, cytokines and mitochondrial transfer. To assess whether mitochondria alone could recapitulate these platelet-associated supportive effects, we compared the impact of platelets and p-mito on HDFs in scratch migration and cell-cycle assays.

**Figure 5:**
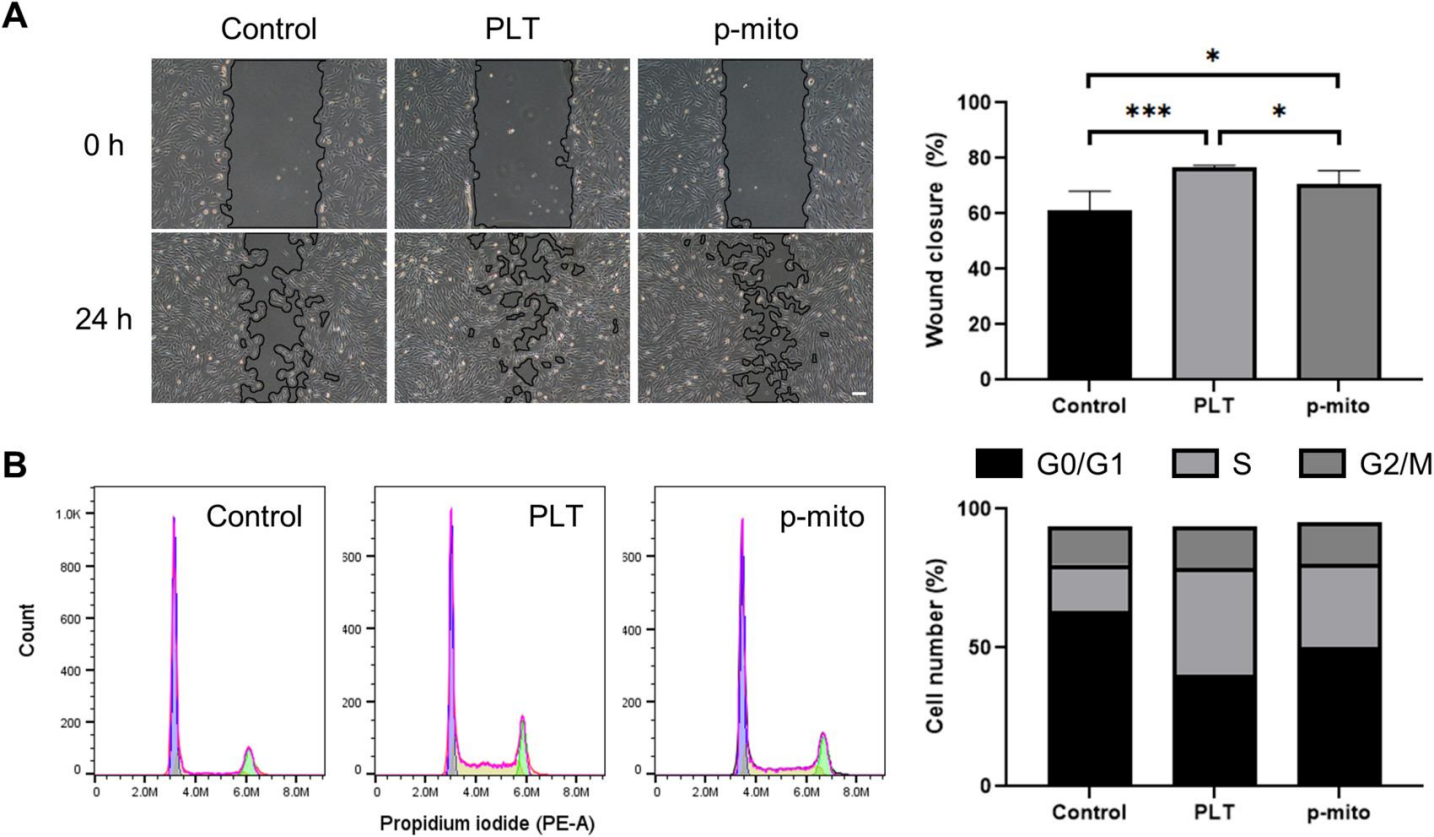
Comparative effects of platelets and plasma-derived mitochondria on fibroblast migration and proliferation. (**A**) Representative images and quantification of wound closure in HDFs at 0 h and 24 h following treatment with control, platelet (PLT) or p-mito. Scale bar = 100 µm. (**B**) Flow cytometric analysis of cell-cycle distribution (G0/G1, S, G2/M phases) in HDFs under the same treatment conditions. *P < 0.05, ***P < 0.001.

In the wound closure assay, p-mito treatment significantly accelerated migration compared with control, reaching levels comparable to platelet treatment (Figure 5A). Cell-cycle analysis showed that both platelets and p-mito increased the proportion of cells entering S phase, and both exceeding control levels (Figure 5B).

Within the limits of these assays, mitochondria were sufficient to reproduce a substantial portion of the platelet-associated response *in vitro*, and thus, represent a principal candidate active component; this does not exclude contributions from other platelet constituents. Given that p-mito can be isolated without platelet activation, avoiding variability associated with growth factor mixtures, they may represent a safer and more standardized approach for potential applicability in cell-based tissue repair therapies.

## Discussion

Our results support mitochondria as a principal candidate active component of platelet-based therapies for the two experimental measures assessed in our *in vitro* models. In THP-1–derived macrophages, mitochondrial preparations induced an M2-associated transcriptional profile within our marker set, and in HDFs, mitochondrial preparations accelerated scratch-wound closure and increased S-phase entry relative to untreated controls. However, this does not imply that mitochondria constitute the sole active component of platelet products. Systematic fractionation and depletion studies will be required to delineate the relative contributions of mitochondrial versus non-mitochondrial platelet constituents [25, 29]. Emerging evidence shows that the metabolic hallmarks of functional mitochondria, including preserved membrane potential and oxidative phosphorylation (OXPHOS), are essential to drive anti-inflammatory macrophage polarization and tissue repair [30, 31]. Moreover, p-mito biased M0 toward an M2-associated profile *in vitro*, consistent with the notion that mitochondria may “educate” macrophages through sustained metabolic/signaling cues, within the constraints of our model and markers [32]. Collectively, these results refine the PRP mechanistic model by highlighting mitochondria as an important candidate effector alongside soluble cytokines and other platelet components, and position p-mito as a promising cell-free modality for macrophage-mediated immune modulation and tissue repair.

Platelet-derived signals exert distinct effects depending on their nature: when platelets were added to M0 THP-1 macrophages in the presence of extracellular calcium, they rapidly triggered M1 polarization (Figure 1). Soluble mediators provoked this acute M1 response, whereas intact mitochondria fostered M2 polarization (Figure 2). This contrast underscores that mitochondrial functionality, rather than mere presence, governs immune fate. Indeed, disruption of mitochondrial membrane potential with CCCP fully abolished the M2-skewing effect, affirming the indispensable role of mitochondrial integrity in immune modulation and cellular fate decisions [33, 34]. In contrast, a growing body of work indicates that intact, functional mitochondria transferred to myeloid cells can reprogram metabolism toward OXPHOS and fatty acid oxidation (FAO), thereby favoring M2-associated states or resolution phenotypes; conversely, loss of membrane potential undermines these effects [30, 31]. This literature aligns with our finding that CCCP-depolarized mitochondria abolished the M2-skewing we observed, supporting a model where mitochondrial functionality, rather than mere presence, modulates immune fate within our assay constraints [30].

Transferred mitochondria appear to impart enduring programming onto recipient macrophages. We observed a two-stage polarization: an initial M1 activation followed by a sustained M1-to-M2 conversion over two weeks, even after removal of extracellular cues (Figure 3). This trajectory suggests that mitochondrial transfer may imprint metabolic or epigenetic memory, resonating with emerging studies showing how mitochondrial integrity and oxidative metabolism can durably shape immune phenotypes [33, 35–40]. Moreover, donor-to-recipient mitochondrial transfer via microvesicle/extracellular-vesicle routes has been shown to enhance macrophage bioenergetics and effector functions; likewise, in our assays, functional mitochondria sustained pro-resolving features, whereas depolarized mitochondria did not [30, 41, 42]. Mechanistically, links between respiration and epigenetic remodeling provide a plausible basis: restoring OXPHOS increases histone acetylation and chromatin accessibility in lesional macrophages, facilitating transcriptional reprogramming [43]. From the perspective of trained immunity, persistent metabolic–epigenetic rewiring can imprint long-lived innate immune memory, typically lasting months in experimental systems, supporting our inference of enduring programming, while acknowledging that such memory can be pro- or anti-inflammatory depending on stimulus, timing and mitochondrial integrity [44, 45]. Thus, mitochondria serve not only as immediate bioenergetic contributors but also as long-term reprogramming agents.

Because our analyses were restricted to RNA-level expression of four surface markers in THP-1–derived macrophages, the findings represent only a limited snapshot of macrophage polarization and should not be interpreted as evidence of full functional repolarization. Additional protein-level assays, cytokine profiling, ROS/phagocytosis measurements and validation in primary human monocyte-derived macrophages will be required to establish whether the observed trends translate into functional anti-inflammatory programming. Moreover, reliance on a single THP-1 cell system limits generalizability, as primary human macrophages display substantially broader phenotypic diversity and context-dependent polarization behaviors.

Safety concerns further differentiate PLT-mito from p-mito, despite both inducing robust M2 polarization without mtDNA-driven inflammatory activation (Figure 4) [46–48]. Minor lysosomal and cytoskeletal protein contamination was detected in PLT-mito preparations (Supplementary Figure S2); while this did not impair *in vitro* immunoregulatory function, such impurities could pose risks in inflamed tissues. Consistent with these concerns, recent work in oncology and autoimmunity shows that activated platelets drive platelet–leukocyte aggregation and NETosis, amplifying thromboinflammation and immune dysregulation, whereas nanodrug strategies targeting the platelet–leukocyte axis can mitigate these sequelae [49, 50]. In contrast, p-mito preparations exhibited minimal non-mitochondrial components, suggesting a superior safety profile for translational use. Importantly, unlike PRP injections, which often cause transient pain and swelling due to early pro-inflammatory mediator release and M1 recruitment [51, 52], direct mitochondrial delivery could limit the extent of this early inflammatory phase and more rapidly establish an M2-dominant environment that promotes anti-inflammatory signaling, angiogenesis, extracellular matrix remodeling and stem cell recruitment [32]. This strategy may be especially valuable in chronic tendinopathies, degenerative joint disease and nonhealing wounds where unresolved inflammation impedes repair.

Stability of p-mito is another critical factor for clinical application. While most preservation strategies rely on cryoprotective sugars such as trehalose or sucrose to maintain mitochondrial integrity during freezing [53], we have developed a proprietary sugar-free buffer that enables long-term cold storage at 4°C. In our *in vitro* assays, p-mito preserved in this solution retained functional activity for up to 12 months (data not shown). This freeze-free approach offers practical advantages for clinical handling and scalability, eliminating the risks associated with freeze–thaw cycles. Ongoing animal studies in degenerative chronic inflammation models are directly comparing freshly isolated and stored p-mito to assess whether long-term preserved mitochondria maintain equivalent therapeutic efficacy [54].

Limitations of this study include exclusive use of THP-1 macrophage cell lines, which may not reflect the full heterogeneity of primary human macrophages. Mechanistic insights were largely derived from surface marker analyses; comprehensive characterization through transcriptomic, metabolic and functional assays is warranted. Validation in primary human macrophages and *in vivo* systems is crucial to determine whether p-mito can consistently induce M2 polarization among diverse immune cell populations within complex tissue microenvironments. While our data indicate that the observed effects depend on mitochondrial integrity, we did not directly quantify the functional status of transferred mitochondria inside macrophages. Future studies will track intracellular mitochondrial function over extended periods (e.g., cellular ATP, and OCR/ECAR at multiple time points) to determine the persistence and magnitude of mitochondrial contributions after uptake. Finally, rigorous *in vivo* evaluation of immunogenicity, biodistribution and long-term safety is essential to advance p-mito toward clinical translation.

## Conclusion

In our *in vitro* THP-1 model, p-mito preferentially supported M2-associated marker expression and appeared to lessen early M1-skewed responses compared with platelet-based treatments. These conclusions are limited to our RNA-level readouts and will require validation in primary macrophage systems. Unlike PLT-mito, which require *ex vivo* activation and complex processing, p-mito can be rapidly obtained from autologous plasma by simple centrifugation while maintaining comparable immunomodulatory potency. This minimal-manipulation approach reduces manufacturing complexity and minimizes inflammatory risk. In addition, the ability of p-mito to create an M2-dominant environment for tissue repair highlights its regenerative potential. Beyond serving as a standardized, point-of-care mitochondrial therapeutic, p-mito may offer broad applicability across chronic inflammatory and degenerative conditions where conventional PRP is limited by transient pain and early inflammatory priming. These findings underscore the clinical potential of p-mito as a next-generation alternative for safe and effective immune-modulatory interventions, with possible relevance to regenerative medicine.

## Supplementary Material

rbaf132_Supplementary_Data
