# Supplementary material for: Plasma-derived mitochondria as a minimal manipulation alternative for attenuating inflammatory immune responses
Source: Regen Biomater. 2025 Dec 26;13:rbaf132. doi: 10.1093/rb/rbaf132 (PMC12883867; doi:10.1093/rb/rbaf132)
Supplement: rbaf132_Supplementary_Data [file rbaf132_supplementary_data.docx]

**[Supplementary File]**

**Plasma-derived Mitochondria as a Minimal Manipulation Alternative for Attenuating Inflammatory Immune Responses**

Seong-Hoon Kim^1^, Eun-Seo Back^2^, Ikhyun Lim^1^, Mina Lim^1^, Mi Jin Kim^3^, Kyunghoon Min^4^, Chang-Koo Yun^3,*^, Yong-Soo Choi^1,2,3,*^

^1^Department of Bio-convergence Science, Graduate School, CHA University, Seongnam 13488, Republic of Korea

^2^Department of Life Sciences, Graduate School, CHA University, Seongnam 13488, Republic of Korea

^3^Department of Life Sciences, CHA University, Seongnam 13488, Republic of Korea

^4^Department of Rehabilitation Medicine, CHA Bundang Medical Center, CHA University School of Medicine, Seongnam 13496, Republic of Korea

*Corresponding authors. E-mail: ckyun@cha.ac.kr (C.K. Yun); yschoi@cha.ac.kr (Y.-S. Choi)


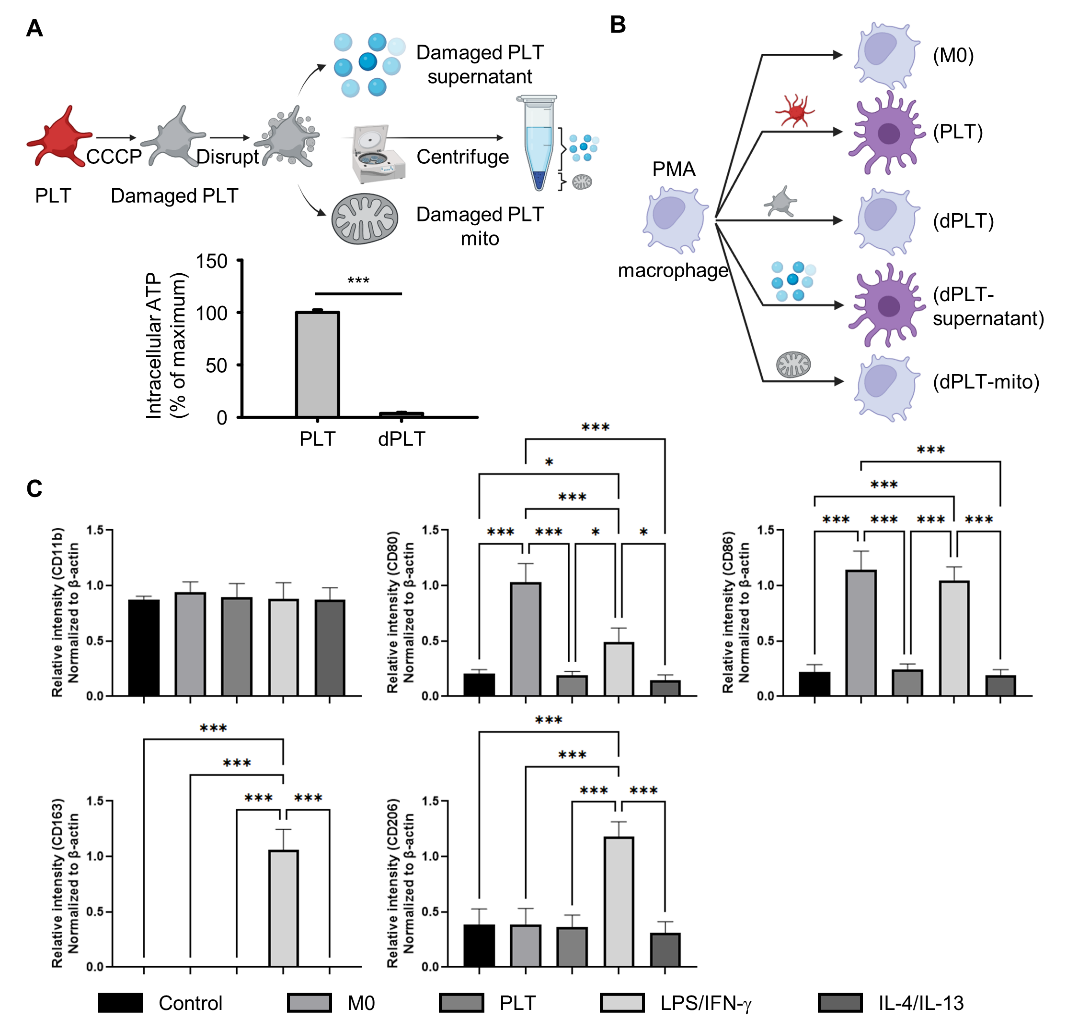


**Supplementary Figure S1. Effects of mitochondrial damage in platelets on macrophage polarization.**

(A) Schematic of generating damaged platelets (dPLT) by CCCP treatment and disruption, followed by fractionation into a mitochondrial pellet (dPLT-mito) and a soluble supernatant (dPLT-supernatant). Lower panel: intracellular ATP levels in intact platelets (PLT) versus dPLT (n = 3). (B) Experimental design for treating PMA-differentiated THP-1 macrophages (M0) with intact PLT, dPLT, dPLT-supernatant, or dPLT-mito. (C) Marker analysis after treatment. Data are expressed as normalized RT-PCR band intensities. *p < 0.05, ***p < 0.001 (n = 3).

**
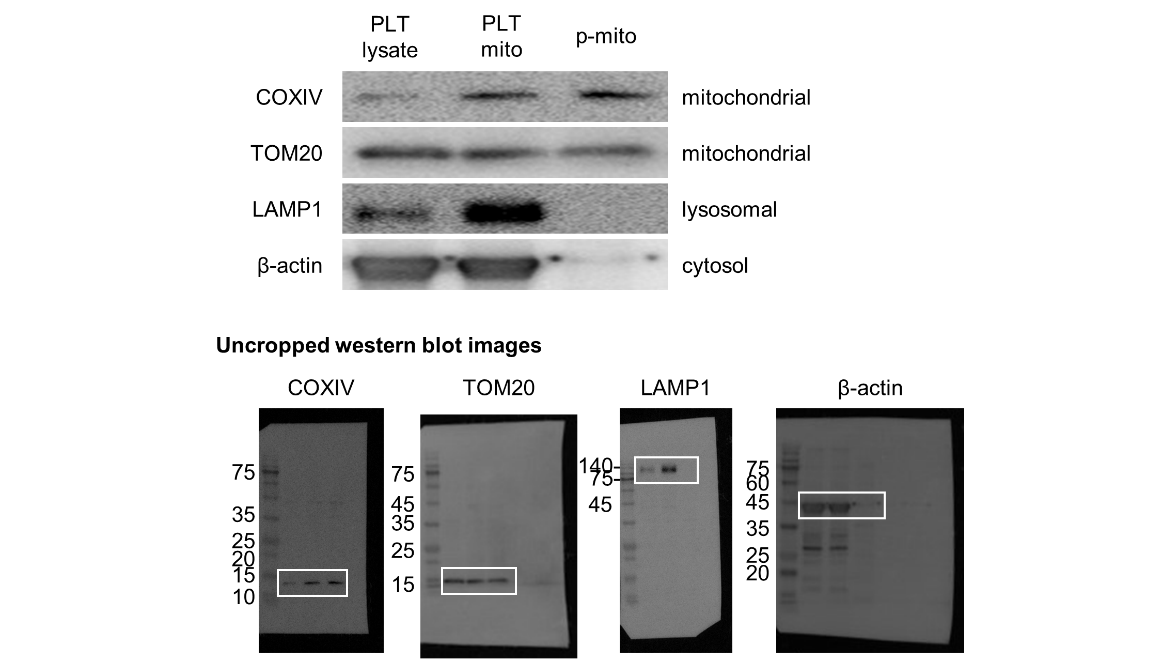
**

**Supplementary Figure S2. Purity assessment of PLT-mito and p-mito preparations.**

Western blot analysis of mitochondrial (COX IV, TOM20), lysosomal (LAMP1), and cytoskeletal (β-actin) proteins in platelet lysate, PLT-mito, and p-mito fractions. Uncropped Western blot images are shown below.

**
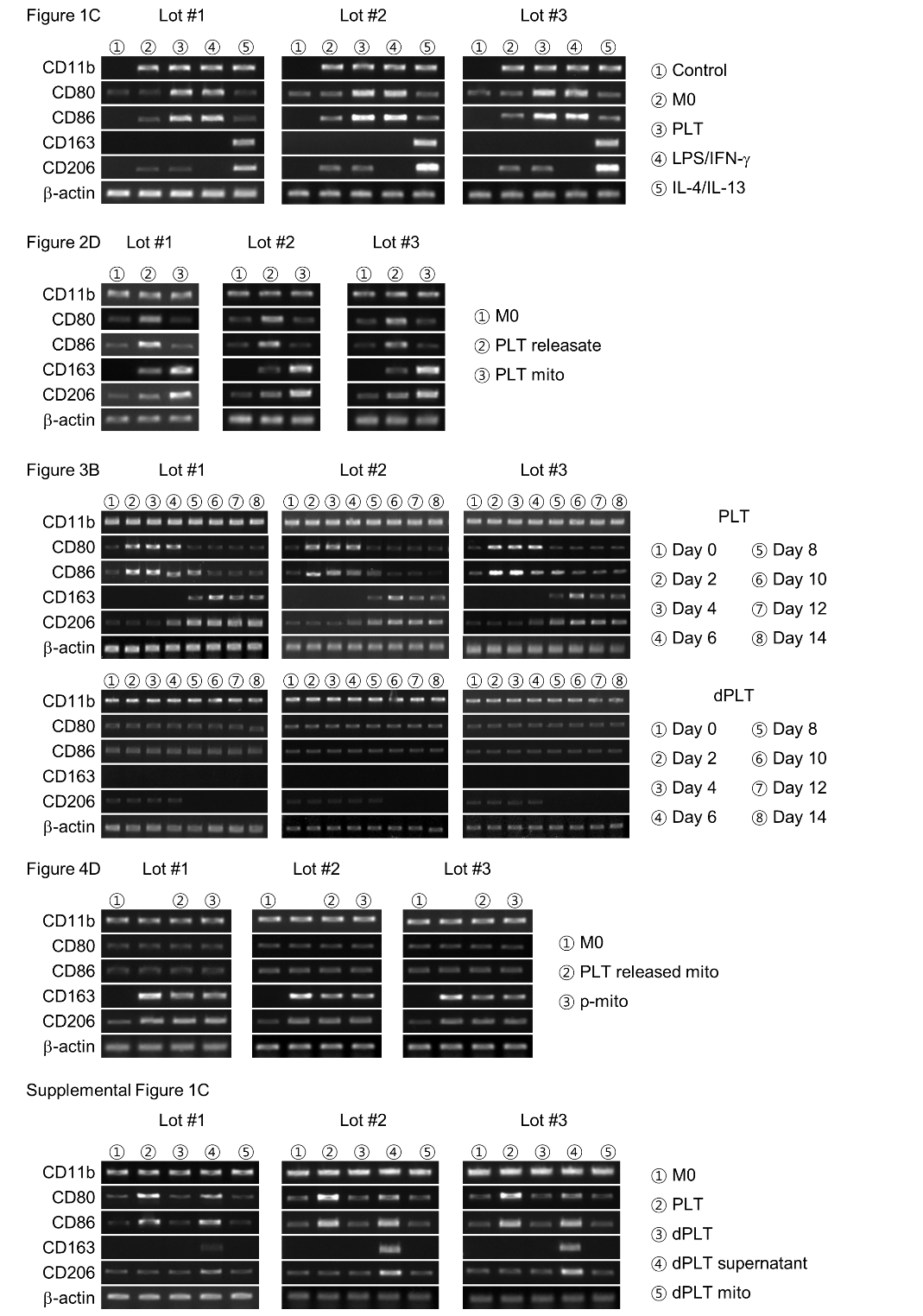
**

**Supplementary Figure S3. PCR gel band images corresponding to Figure 1C, 2D, 3B, 4D, and supplementary Figure 1C.**

**Supplementary Table 1. Individual donor-level data for p-mito yield and function.**

These data were reused and reorganized from Lim et al., “Plasma-derived Mitochondrial Transplantation Attenuates Paraspinal Muscle Atrophy Following Spinal Surgery,” Regenerative Biomaterials, currently in press. The group-average values were previously published; here, individual donor-level data are added for transparency and reproducibility.

| Donor ID | Sex | Age | Health status | p-mito yield  (μg/mL) | ATP synthesis  (nM) |
| --- | --- | --- | --- | --- | --- |
| S05 | F | 40 | Healthy | 2.229 | 109 |
| S06 | M | 31 | Healthy | 2.197 | 102 |
| S07 | M | 54 | Hyperlipidemia | 2.048 | 106 |
| S08 | M | 53 | Cholecystectomy, Hyperlipidemia | 2.838 | 91 |
| S09 | F | 36 | Healthy | 2.309 | 98 |
| S10 | M | 28 | Ankylosing spondylitis | 1.175 | 113 |
| S11 | F | 34 | Healthy | 2.628 | 105 |
| S12 | M | 31 | Healthy | 2.585 | 99 |
| S13 | F | 59 | Healthy | 2.654 | 117 |
| S14 | F | 46 | Healthy | 2.389 | 86 |
| S15 | F | 29 | Healthy | 1.899 | 124 |
| S16 | F | 23 | Healthy | 1.966 | 119 |
| S17 | F | 42 | Healthy | 2.341 | 123 |
| S18 | F | 37 | Essential hypertension | 2.237 | 101 |
| S20 | M | 44 | Healthy | 1.894 | 101 |
| S21 | M | 27 | Healthy | 2.42 | 105 |
| S24 | M | 59 | Healthy | 2.176 | 102 |
| S25 | F | 50 | Healthy | 2.231 | 105 |
| S26 | F | 62 | Hypertension | 2.177 | 106 |
| S27 | F | 29 | Healthy | 2.329 | 101 |
| S28 | F | 42 | Healthy | 2.142 | 98 |
| S29 | M | 30 | Healthy | 2.468 | 103 |
| S30 | F | 48 | Healthy | 2.314 | 102 |
| S31 | F | 60 | Sjogren's syndrome | 2.206 | 99 |
| S32 | F | 58 | Healthy | 2.182 | 106 |
| S33 | M | 45 | Healthy | 2.232 | 101 |
| S34 | M | 42 | Healthy | 2.149 | 97 |
| S35 | F | 26 | Healthy | 2.226 | 109 |
| S36 | F | 24 | Healthy | 2.462 | 104 |
| S37 | F | 31 | Healthy | 2.284 | 102 |
| S38 | F | 31 | R/O) Fibromyalgia, multiple sites | 2.392 | 105 |
| S39 | M | 30 | Healthy | 1.996 | 102 |
| S40 | F | 27 | Healthy | 2.095 | 106 |
| S41 | M | 30 | Healthy | 2.292 | 97 |
| S42 | F | 27 | Healthy | 2.015 | 97 |
| S43 | F | 32 | Hyperthyroidism | 2.409 | 104 |
| S44 | F | 45 | Healthy | 2.054 | 99 |
| S45 | M | 19 | Healthy | 2.336 | 109 |
| S46 | M | 19 | Healthy | 2.088 | 102 |
| S47 | F | 34 | Healthy | 2.486 | 105 |
| S48 | F | 43 | Healthy | 2.272 | 107 |
| S49 | F | 70 | Diabetes mellitus, Hyperlipidemia | 2.125 | 97 |
| S50 | M | 73 | Cerebral infarction | 1.995 | 107 |
| S51 | F | 37 | Healthy | 2.419 | 103 |
| S52 | F | 42 | Healthy | 2.495 | 104 |
| S53 | F | 47 | Healthy | 2.261 | 101 |
| S54 | F | 27 | Healthy | 2.044 | 96 |
| S55 | F | 41 | Healthy | 2.292 | 101 |
| S56 | F | 36 | Healthy | 2.477 | 101 |
| S57 | M | 47 | Healthy | 2.383 | 105 |
| S58 | M | 44 | Healthy | 2.287 | 109 |
| S59 | M | 83 | Post laminectomy syndrome | 1.374 | 104 |
| S60 | M | 67 | Arrhythmia,  Total laminectomy of T11-L2 vertebrae | 2.064 | 108 |
